# Impact of ER Stress and ER-Mitochondrial Crosstalk in Huntington’s Disease

**DOI:** 10.3390/ijms23020780

**Published:** 2022-01-11

**Authors:** Shuvadeep Maity, Pragya Komal, Vaishali Kumar, Anshika Saxena, Ayesha Tungekar, Vaani Chandrasekar

**Affiliations:** Department of Biological Sciences, Birla Institute of Technology and Science (BITS)-Pilani (Hyderabad Campus), Shameerpet-Mandal, Hyderabad 500078, Telangana, India; pragya@hyderabad.bits-pilani.ac.in (P.K.); p20200006@hyderabad.bits-pilani.ac.in (V.K.); h20201290014@hyderabad.bits-pilani.ac.in (A.S.); h20201290002@hyderabad.bits-pilani.ac.in (A.T.); h20201290001@hyderabad.bits-pilani.ac.in (V.C.)

**Keywords:** ER stress, Huntington’s disease (HD), ER, mitochondria, mitochondria associated ER membranes (MAM)

## Abstract

Accumulation of misfolded proteins is a common phenomenon of several neurodegenerative diseases. The misfolding of proteins due to abnormal polyglutamine (PolyQ) expansions are linked to the development of PolyQ diseases including Huntington’s disease (HD). Though the genetic basis of PolyQ repeats in HD remains prominent, the primary molecular basis mediated by PolyQ toxicity remains elusive. Accumulation of misfolded proteins in the ER or disruption of ER homeostasis causes ER stress and activates an evolutionarily conserved pathway called Unfolded protein response (UPR). Protein homeostasis disruption at organelle level involving UPR or ER stress response pathways are found to be linked to HD. Due to dynamic intricate connections between ER and mitochondria, proteins at ER-mitochondria contact sites (mitochondria associated ER membranes or MAMs) play a significant role in HD development. The current review aims at highlighting the most updated information about different UPR pathways and their involvement in HD disease progression. Moreover, the role of MAMs in HD progression has also been discussed. In the end, the review has focused on the therapeutic interventions responsible for ameliorating diseased states via modulating either ER stress response proteins or modulating the expression of ER-mitochondrial contact proteins.

## 1. Introduction

Nearly 35 years after its discovery, Huntington’s disease (HD) is one of the neurological disorders with no cure. It is an autosomal dominant neurodegenerative disease with progressive motor and cognitive impairments. Despite its well-studied genetic route, the cellular mechanisms of the disease onset and progression are highly complex, involving several factors that lead to gain of toxicity. The critical neuropathology in HD is the preferential loss of medium spiny neurons at the striatum [1]. At the molecular level, the cause of HD is the CAG trinucleotide repeat expansion within exon 1 of the human HD gene. Translation of mutated gene translated to mutant huntingtin(mtHTT) protein with polyglutamine repeats [2]. CAG repeats (>35 CAG thought to be more toxic, PolyQ) at the N-terminus make mHtt highly aggregation prone [3,4]. Accumulation of cytoplasmic aggregates and nuclear inclusions throughout the brain is one of the major characteristics of HD pathology. Initial studies showed polyglutamine inclusions could sequester numerous other proteins contributing to the loss of function phenotypes. Initial investigations suggested the toxic nature of small oligomers are their sequestering nature of other proteins necessary for transcription and protein quality control [5]. Moreover, this aggregate formation is a highly complex multi-step process that depends on a range of factors such as amino acid sequences in the flanking regions of polyglutamine stretches, post-translational modifications, presence of the molecular chaperones, etc. [6,7,8]. Additionally, it was found that soluble oligomers majorly cause the cytotoxicity in HD, and these oligomers are frequently bound to proteins with low-complexity sequences, while insoluble inclusions are less interactive and strongly associated with protein quality control components (e.g., Hsp40 chaperones and factors of the ubiquitin-proteasome system) [9,10]. The mHtt oligomers inhibit endoplasmic reticulum (ER)-associated degradation (ERAD) and induce endoplasmic reticulum (ER) stress [11]. Here, in this review, we aim to highlight some recent HD research findings focusing on the involvement of ER and ER stress response pathways. We will also extend the discussion about the impact of proteins involved in ER-mitochondrial communication over HD. In the end, we have addressed how different therapeutic approaches targeting ER stress pathway proteins could be effective in HD pathology.

## 2. ER and ER Stress Response

**Figure 1:**
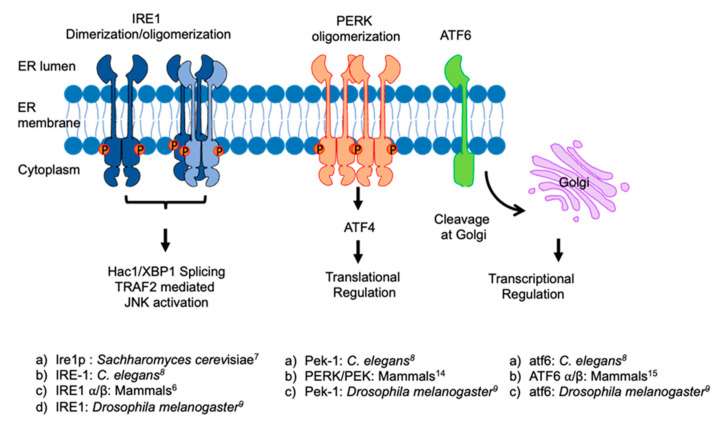
ER stress sensors are conserved from yeast to mammals. List (below) shows ER stress sensors in various model organisms. Numbers (in prefix) indicate the reference of the research articles.

ER is a continuous and dynamic membranous organelle. The intricate network spreads over the cells and also shows specialized dynamics depending on cell types [12,13]. This is the major site for membrane and secretory protein synthesis [14,15]. Besides protein synthesis, it serves as a major site for lipid synthesis and calcium storage. The unique environment of the ER is not only ideal for protein and lipid synthesis but also facilitates its modifications. As a major protein synthesis and folding site, it balances the protein folding and misfolding related biological events to maintain protein homeostasis. Disturbances in the ER function and its integrity due to physiological, pathological, or environmental conditions lead to ER stress. ER stress activates a series of beneficial transcription and translation events, collectively referred to as Unfolded protein response of ER (UPR^ER^) [16]. In higher organisms, the UPR^ER^ pathway consists of three separate branches and is controlled by three different sensor proteins present at the ER membrane (Figure 1). The yeasts have only one sensor protein i.e., Ire1 [17]. Each of the sensor proteins harbors a transmembrane helix and a luminal domain. The luminal domain senses the accumulation of misfolded proteins through direct or indirect interactions [18,19,20,21,22,23]. Lipid disequilibrium can also lead to activation of the UPR sensors. Inositol, requiring protein 1 IRE1 (IRE1 in mammals, IRE-1 in Caenorhabditis elegans, IRE1 in *Drosophila*, and Ire1p in *Saccharomyces cerevisiae*) [24,25,26,27], is a ER localized transmembrane protein which undergoes homodimerization or oligomerization followed by trans-autophosphorylation when misfolded proteins accumulate inside ER. Upon dimerization/oligomerization of Ire1, the RNAse domain of the cytosolic part of IRE1 initiates the unconventional splicing of mRNA and the spliced mRNA is translated to an active transcription factor (XBP1 in mammals, XBP-1 in *C. elegans*, and Hac1p in *S. cerevisiae*) [28]. This transcription factor increases the genes encoding proteins that help in the mediates expression of protein degradation, protein folding, and lipid metabolism. Moreover, Ire1 Kinase domain catalyzes the transphosphorylation reaction which led to the disassembly of Ire1 signaling complexes and is a critical component of the UPR homeostatic feedback loop [29]. Additionally, IRE1 also reduces the protein-folding load by controlling the regulated Ire1-dependent decay (RIDD) of specific sets of mRNA [30]. RIDD is conserved in higher eukaryotes and found in fission yeast. Fission yeast (*Schizosaccharomyces pombe*) lacks the Hac1 transcriptional pathway. Interestingly, these two functions (Hac1/xbp1 signaling and RIDD) are controlled through two different modes of IRE1 action. Cooperative activity of IRE1 subunits is essential for HAC1/XBP1 splicing. RIDD activity by IRE1 is distinct from cleavage of XBP1/HAC1 intron by IRE1. Though both activities use the same catalytic residues, they have distinct substrate binding sites [31]. Xbp1/Hac1 predominantly activates signaling that helps cell survival, while RIDD leads to cell death.

Another important branch of UPR^ER^, which is conserved in higher organisms (absent in yeast), is protein kinase RNA-like ER kinase (PERK in mammals, PEK-1 in C. elegans, and Pek-1 in Drosophila) [26,27,32]. This branch is the main UPR arm that can control the global translation inhibition via eukaryotic translation initiation factor 2 alpha (eIF2alpha). Like IRE1, homodimerization of PERK leads to phosphorylation of eIF2alpha which, in turn, induces a global down regulation of cap dependent translation. Interestingly, cap independent translations of specific mRNA species escape global translation downregulation, including the activation of transcription factor 4 (ATF4 in mammals and ATF-4 in *C. elegans*) [33]. ATF4 regulates the upregulation of ER stress response genes controlling integrated stress response [34]. Besides the metabolic and translational control, ATF4 can activate apoptosis during prolonged ER stress via upregulation of CCAAT enhancer binding protein (C/EBP) homologous protein (CHOP) [34,35].

The third branch of UPR^ER^ is activated after the proteolytic cleavage of an ER-resident protein, which translocates to the Golgi under ER stress to become a proteostasis-promoting transcription factor, activating transcription factor 6 (ATF6 in mammals, atf6 in *C. elegans* and *Drosophila*) [26,27,36]. ATF6 is a 90-kDa ER transmembrane protein (p90ATF6) with evolutionary conserved N-linked glycosylation sites within its luminal domain. ER stress induces transit of p90ATF6 from the ER to the Golgi where it is cleaved by the S1P/S2P protease system to generate a nuclear form p60ATF6 that acts as a transcriptional activator. p60ATF6, after its relocation to the nucleus, activates the transcription of a wide variety of ER stress-inducible promoters including Grp78/BiP, protein disulfide isomerase (PDI), and CHOP [36,37]. The glycosylation status of p90ATF6 may serve as a sensor for ER homeostasis, resulting in ATF6 activation to trigger the UPR [38,39,40].

The extended function of the ER and stress response pathway is related to the degradation of those proteins that are beyond repair. Those terminally misfolded proteins are transported from ER and targeted to proteasomal degradation through ERAD. In yeast, Hrd1p, and Doa10p, ER transmembrane protein ligases recognize misfolded proteins and tag (polyubiquitination) them [41,42]. The polyubiquitinated misfolded proteins are guided to cytosol with the help of ERAD machinery Cdc48p (p97 or valosin-containing protein in humans) for degradation via proteasome [43]. Dysregulation of the UPR^ER^ is directly or indirectly connected to several complex disorders, including neurodegenerative diseases. In the below sections, we will further discuss the relation of these three ER stress response pathways with HD pathology.

## 3. ER Stress Proteins and Their Involvement in HD Pathology

Undoubtedly, protein misfolding stress of ER is connected to age and age-related disorders. During aging, it is known that UPR^ER^ becomes dysregulated across multiple organisms. For example, in aged mice there is a notable decline of ER quality control proteins [44,45]. Like other neuronal diseases, HD is also linked to ER stress. While mutant Htt (mHtt) protein is known to be involved in pathogenesis, the wild type Htt (Huntington) is required for ER-Golgi secretory transport and secretory vesicle fusion at the plasma membrane. Htt is a 3144 amino acids long membrane-associated scaffolding protein. It associates with endocytic and exocytic vesicles modulating their trafficking along cytoskeletal tracks. Studies found homozygous knock in of 140Q in mice showed defects in secretory pathways. Interestingly, heterozygous fibroblasts from a Huntington’s disease patient with a 180Q expansion did not display defects in the early secretory pathway [46]. Recent advances also explored involvement of other pathways interacting with ER stress pathways. This evidence suggests a complex repertoire of signaling events that is underlying beyond its genetic involvement of mHtt.

### 3.1. IRE1

The link of ER stress and HD was initially observed when STHdhQ111 striatal cell lines harboring full-length mutant Huntingtin showed an abnormal expansion of perinuclear ER [47]. The expansion of ER is one of the hallmarks of ER stress. This study began a new concept that linked ER stress and HD pathology. A subsequent study investigating the cause of cell death in PC12 and related neuronal cell lines harboring PolyQ found that IRE1 activated TRAF2-ASK1 complex formation and activation of c-Jun N-terminal kinase (JNK) signaling plays an important role in striatal neuronal death. Interestingly, they observed that ASK1−/− mouse neurons were resistant to polyQ-induced ER stress, proteasome dysfunction, JNK activation, and cell death [48]. Subsequent studies using yeast and PC12 cells expressing expanded polyglutamine clearly showed inhibition of ERAD [11,49]. Indication of the involvement of IRE1 branch was established when Carnemolla et al. found upregulation of ER stress marker mRNAs, namely BiP, CHOP, and Herpud1, in parietal cortex in HD postmortem brains compared to control [50]. Additionally, this group, for the first time, demonstrated that ER stress itself is a very early event in HD pathogenesis detected in the striatum of Hdh knock-in mice before the formation of any visible aggregates. They identified an ER stress related marker, Rrs1, showed increased expression as a pre-symptomatic event [50]. Korhonen’s group found the expression of N-terminal huntingtin proteins with expanded polyglutamine (polyQ) repeats causes cell death in neuronal PC6.3 cells through downregulation of NFkB via IRE1-TRAF2 ER stress pathway [49,51]. Further dissection of the IRE1-TRAF2 pathway showed increased accumulation of mtHTT is due to inhibition of autophagic flux. In HD mouse models and in HD patients, p-IRE1(ER stress marker) and p62 (autophagy markers) were upregulated exclusively in the striatal tissues. Additionally, downregulation or IRE1 expression rescued rough-eye phenotype by mtHTT in a HD fly model [52]. IRE1 splices XBP1, which acts as a transcription factor to induce activation of UPR related downstream genes. Interestingly, XBP1 deficient mice were found to be more resistant to developing HD features and showed a drastic decrease in mtHTT levels. Again, the XBP1 deficiency leads to enhanced expression of Forkhead box O1 (FoxO1) which, in turn, upregulates autophagy. This study specifically established the unique role of XBP1 transcription factor beyond its role in ER stress. In XBP1 deficient cells, FoxO1 transcription factor upregulated. FoxO1 is a key transcription factor regulating autophagy in neurons. Thus, upregulation of FoxO1 induces autophagy. The enhanced autophagy is beneficial for the removal of aggregates. [53].

Gradual understanding on mtHTT aggregates found that not the aggregated form but the soluble oligomeric polyglutamine-expanded huntingtin itself is cytotoxic, and the pathogenic huntingtin oligomers inhibits ERAD and induces all the three branches of ER stress (IRE1, PERK, and ATF6) before its visible aggregation in terms of inclusion bodies [10]. It also demonstrated that the overexpression of p97/VCP can reduce ER stress, indicating sequestration of the oligomers ameliorates. VCP/p97 can regulate Beclin-1-dependent autophagy initiation process [54]. This study thus supports the complex balance between ER stress pathways with autophagic pathways. It was also reported, VCP/P97 can act as disaggregase against the toxic Huntingtin-exon1 aggregates [55].

The relationship of the formation of mHtt deposits, i.e., inclusion bodies (IBs) and neurodegeneration, is complex. Earlier studies found a positive correlation of visible inclusion bodies with neurodegeneration [56,57]. Interestingly, no correlation or opposite correlation was also observed by other groups [58,59]. This is probably because other multimeric complexes co-exist with IBs. Thus, low temporal and spatial resolution of the aggregates limits our understanding and influences the interpretation. HD pathogenic toxicity from soluble oligomeric forms or the inclusion body has emerged as an important question in context of the HD pathogenicity. To answer HD related toxicity of different aggregates, Arrasate et al. (2014) demonstrated microscopic inclusion body formation by mtHTT aggregates was protective and beneficial as it helped in coping the response to toxic mutant huntingtin [60]. Large protein aggregates of the mutant Huntingtin protein (mHTT) are, thus, protective because it can be degraded by macroautophagy (MA), which encloses aggregates in double-membraned autophagosomes that then fuse with lysosomes for degradation [61]. Again, IRE1 plays an interesting role in controlling the mHTT aggregate formation. Retrograde trafficking helps the localization of aggregated proteins to the microtubule organizing center (MTOC) and disruption in trafficking hinders the degradation of these aggregates. A very recent preprint found how degradation of Blos1 mRNA via IRE1 mediated process influences mHtt degradation prior to the formation of large aggregates [62]. Bae et al. showed the retrograde trafficking and clustering of lysosomes and late endosomes (LEs) near the MTOC is induced by the IRE1. Moreover, expression of the aggregation-prone Huntingtin protein induces IRE1 mediated targeted degradation or RIDD of BLOS1 mRNA. As a member of BLOC1-related complex (BORC), BLOS1 is known to be involved in trafficking of lysosomes toward the periphery of the cells [63]. Earlier, it was reported that mHTT aggregates can be degraded by macroautophagy [64]. During ER stress, degradation of the BLOS1 via RIDD helps the repositioning of late endosome/lysosome to the microtubule organizing center (MTOC) and leads to enhanced degradation of ubiquitinated proteins and protects cells from apoptosis. A detailed study by Bae et al. reported that larger aggregates of mHTT potentially degrade by macroautophagy. However, prior to formation of larger aggregates, mHTT is degraded via an ESCRT (endosomal sorting complex required for transport)-dependent, endosomal microautophagy pathway. This pathway is controlled by Blos1 degradation and appears to protect cells from a toxic, less aggregated form of mHTT [62].

### 3.2. PERK

As it is evident from earlier discussion, crosstalk between ER stress and autophagic pathways strongly contribute to HD pathology. Like IRE1, the PERK branch is also found to be important, and it also regulates autophagy formation. It is already established that phosphorylation of eIF2α inhibits ER-stress-induced caspase-12 activation and ER-stress-mediated cell death [65]. Using an in vitro cellular model, Kouroku et al. (2007) found PolyQ72, but not PolyQ11, aggregates can induce endoplasmic reticulum (ER) stress-mediated cell death. Expression of PolyQ72 also stimulated Atg5-dependent conversion from LC3-I to -II, a major step for autophagy formation. Interestingly, eIF2α A/A mutation (non-phosphorylated mutant) inhibited polyQ72-induced LC3 conversion and increased the number of cells with PolyQ72-induced caspase-12 activation. This study indicates a probable mechanism of PolyQ72- and ER-stress-induced LC3 conversion, LC3-I to -II, via activation of the PERK/eIF2α pathway [66]. Direct evidence for the involvement of PERK was described when it was found ER stress-induced eIF2-alpha phosphorylation control sensitivity of striatal neurons to pathogenic Huntingtin. Compared to other cells, the level of eIF2α phosphorylation is significantly lower in the striatal cells. Interestingly, the presence of pathogenic mHtt increased the eIF2 alpha phosphorylation in STHdh Q111/111 cells and in the murine striatum [67]. Authors found an intricate balance between the beneficial and harmful effects of eIF2α phosphorylation by PERK. PERK inhibition compensated for pathogenic huntingtin toxicity in striatal neurons [66,68]. In search of a therapeutics for HD, Lederkremer’s group further proceeded with a screening and ended up with another exciting observation. In previous studies [66,68], a PERK inhibitor (A4) reversed the toxicity of mHtt expression in a striatal neuron cell line. However, later, in search of the new compounds, the group discovered MK-28 showed an enhanced protective effect than that of the mother compound A4. Surprisingly, it was found that the compound MK-28 is an activator of PERK instead of an inhibitor [69]. This twist actually makes PERK as a central modulator of survival of cells during HD balancing eIF2alpha phosphorylation. Probably PERK level itself matters in the case of mtHTT pathology. Importantly, the MK-28 induced beneficial effect propagated exclusively through PERK as there is lack of protective effect which was observed in PERK−/− cells. However, the detailed effects of MK-28 on the PERK pathway in vivo should be further explored [69].

### 3.3. ATF6

The ATF6 branch of the ER stress pathway has been involved in various developmental pathways in higher organisms. In comparison with other adult tissues, the glycosylated ATF6 is elevated in the embryonic brain of mice [70]. Low level of ER stress, which does not elicit apoptosis and activation of ATF6, is essential during the differentiation of bone marrow stromal cells into neurons [71]. Interestingly, a striking correlation between maximum ATF6 activation at postnatal day 10 with the onset of myelination was observed [72]. In higher organisms, ATF6 exhibit neuroprotective effects for several diseases [73]. The link between ATF6 and Huntington’s was described by Fernandez et al. (2011). Processing of ATF6 alpha was impaired in HD patients as well as mouse models. The impaired processing leads to decreased levels of the cytosolic N-terminal ATF6α region which acts as a transcription factor. Intriguingly, alterations of ATF6 are accompanied by a decrease in Rheb [74]. These collective alterations force striatal neurons for aberrant re-entry to cell cycle phase. This unusual cell cycle entry and compromised execution of the ER-stress response in the postmitotic neuron could lead to activation of HD pathology [72,75]. Later, a link was found between calcium sensor and ATF6. A neuronal calcium sensor (NSC) DREAM (downstream regulatory element antagonist modulator) controls Ca^2+^ and protein homeostasis. It was found that downregulation of DREAM is beneficial for neuronal survival in the striatum. Motor coordination in R6/2 mice, a model of Huntington’s disease (HD), improved after the downregulation of DREAM. Additionally, downregulation of DREAM also improved ATF6 processing in the hippocampus [72,75,76]. Another study by Wang et al. showed the effect of TD-induced neurodegeneration and noted that ER stress markers, such as GRP78, XBP-1, CHOP, ATF-6, phosphorylated eIF2α, and cleaved caspase-12, have increased rapidly [77].

Pharmacological inhibition of DREAM activity can induce UPR. Thus, derepression of ATF6 signaling and activation of pro-survival effects of UPR is linked via DREAM. Taken together, neuroprotection via ATF6 could be an interesting avenue for HD treatment.

### 3.4. ERAD

The connection between ER stress and PolyQ toxicity has been reported in yeast as well as in PC12 (neuronal cells). These cells exhibit defects in endoplasmic reticulum (ER)-associated degradation (ERAD). This happens due to the entrapment of Npl4, Ufd1, and p97 (essential ERAD proteins) by the mHtt fragments. Defects of ERAD altered ER protein homeostasis contributing to PolyQ toxicity [11]. As described earlier by Leitman et al., soluble oligomeric PolyQ structures are more toxic and cause cytotoxicity by inhibiting ERAD. mHtt affects the ubiquitin-proteasome pathway, thereby hindering cytosolic protein degradation and ERAD. Accumulation of ERAD substrate is not proportional to the amount of visible aggregates of mHTT. The accumulation of ERAD substrate and subsequent ER stress was initiated when mHtt is in its monomeric or oligomeric phase [10]. Another way through which mHtt is interacting with ERAD pathway was interaction via gp78, an ER membrane-anchored ubiquitin ligase (E3) involved in ERAD. mHtt (17Q or 138Q aggregates) interaction competitively reduces polyubiquitinated protein binding to gp78 and blocks the interplay between gp78 and p97/VCP, which is essential for ERAD. This study provided a novel molecular mechanism that supports the involvement of ER stress in HD pathogenesis [78]. mHTT expressing cells are acutely sensitive to misfolded secretory proteins. However, when treated with ER stressors such as DTT or tunicamycin, Lajoie et al. found decreased BiP-GFP mobility due to increasing amounts of misfolded proteins in affected striatal cells elevating UPR activation. mHtt-expressing cells exhibited decreased misfolded protein flux as a result of ERAD dysregulation [79].

Thus, it is evident that all the three branches of the UPR pathway are linked to HD toxicity (Table 1). Moreover, ERAD impairment is one of the major factors for HD toxicity. Overall, protein homeostasis maintenance is crucial to prevent mHtt related toxicity. Thus, modulation of UPR pathway with specific inhibitors or activators could be the most effective therapeutic in the future.

## 4. MAM Proteins and Their Involvement in HD Pathology

The physical interaction between outer mitochondrial membranes with a subregion of endoplasmic reticulum (ER) is referred to as mitochondria associated ER membranes (MAMs). The physical contact between ER and mitochondria becomes one of the major signaling hubs that controls several signaling processes, including Ca^2+^ signaling, lipid metabolism, ER stress signaling, mitochondrial fission/fusion driven process, and autophagy. Strikingly, in neurodegenerative diseases, such as AD and PD, most of the mentioned pathways are found to be altered. Similarly, the ER–mitochondria interface mitochondrial associated proteins (MAM) are also involved in HD pathology. ER stress sensors such as Ire1 and PERK are also considered as MAM components [80]. Apart from these already described players, mitochondrial structural proteins involved directly in the mitochondrial dynamics process (Drp1, Fis1, Opa1) are known to contribute significantly [81,82,83]. Similarly, the Sigma-1 receptor (S1R) is a multi-functional, ligand-operated protein situated at MAM site which has also been associated with various neurological disorders, including amyotrophic lateral sclerosis/frontotemporal dementia, and Alzheimer’s (AD) and Huntington’s diseases (HD) [84,85,86].

### 4.1. Mitochondrial Dynamics Protein (Drp1, Fis1, Opa1, Mfn1)

Mitochondrial dynamics are maintained through fission–fusion events and are closely related to mitochondrial function. Some of the proteins involved in this process are highly conserved from yeast to mammals. Several of these proteins are related to dynamin, a GTPase. Fission is regulated by the GTPases Drp1 and Dyn2, whereas the GTPases Mfn1, Mfn2, and OPA1 executed fusion processes [87]. The dynamin-related protein 1 (Drp1), which is the master regulator of the mammalian mitochondrial fission, is the ortholog of yeast Dnm1p [88]. In mammals, four additional mitochondrial receptors Fis1, Mff, and MIEF1 and MIEF2 (also known as MiD51 and MiD49, respectively) interact with Drp1 to constitute the mitochondrial fission machinery [89,90]. Like fission, the fusion process is also regulated by dynamin-related GTPases. Mitochondrial outer membrane localized Mitofusins (Mfn1 and Mfn2) and the mitochondrial inner membrane protein optic atrophy 1 (OPA1) played significant roles for fusion of mitochondrial outer membranes and inner membranes, respectively [91]. OPA1 is unique in its structure and function as it exists as two forms: inner membrane-anchored long OPA1 (L-OPA1) and its proteolytic cleaved form which lacks transmembrane anchor, called short OPA1 (S-OPA1) [92].

Direct interaction of mHTT with Drp1 plays a significant role in HD development [93]. Increased mitochondrial fragmentation and DRP1 translocation was observed in the primary striatal neurons of YAC128 transgenic mice [94]. Not only in cellular models but in post-mortem HD patient striatum, smaller mitochondria with an increased DRP1 protein and a reduction in the expression of the fusion protein mitofusin 1 were observed in late-stage HD [95]. Further studies using postmortem HD brains and primary neurons from transgenic BAC-HD mice (expressing fl-mhtt with 97 glutamine repeats under the control of endogenous htt regulatory machinery on the bacterial artificial chromosome (BAC)) clearly found increased abnormal mitochondrial dynamics [93,96]. Moreover, direct interaction between mutant Htt and GTPase Drp1 elevated the GTPase Drp1 enzymatic activity resulting in defective anterograde mitochondrial movement and synaptic deficiencies [93,96]. Increased DRP1 activity associated with increased fragmentation of the mitochondrial network has also been reported in human HD fibroblast and lymphoblast cell cultures as well as in HD mouse primary neurons and brain [93,97]. Reduction in Drp1 recruitment to the mitochondria was found to improve mitochondrial structure in the cardiac tissue of R6/2 mice [98].

It has been observed that expression of mHTT is ubiquitous and impairs other organ systems. One of the earliest symptoms during HD is cardiovascular dysautonomia as well as the deterioration of circadian rhythms. Drp1/Fis1-mediated mitochondrial fragmentation plays a significant role in HD related dysfunction of cardiac cells [98]. Drp1 has been found to interact with fission 1 (Fis1), leading to excessive mitochondrial fragmentation and production of reactive oxygen species (ROS) along with lysosomal dysfunction when PolyQ77 was expressed in cardiac models of Huntington’s disease (H9C2 cells) [98]. Reduction in Fis1-mediated Drp1 recruitment to the mitochondria using selective inhibitor, P110 (Drp1 inhibitor), significantly restored the mitochondrial structure in the cardiac tissue of HD R6/2 transgenic mice [98]. Using the same mouse model (R6/2) as earlier, Hering et al. (2017) found an abnormal morphology of mitochondrial cristae in the striatum and cortex. Dysregulated OPA1 oligomerization was found to be a major reason for the abnormal cristae reflecting that mHtt can directly impair the OPA1 oligomerization [99].

### 4.2. Sigma-1 Receptor S1R

S1R resides mainly as a 223-amino-acid-long transmembrane protein localized at MAM and regulates calcium exchange between the two organelles through inositol trisphosphate receptor type 3 (IP3R type 3) [100]. There are cholesterol-enriched microdomains within the ER membrane where S1R can directly interact with cholesterol domains, promoting the formation and stabilization of MAM microdomain [101]. Pull down experiments showed ER resident protein BiP is a major interacting partner of S1R. It forms complexes at MAM with ER chaperone protein, BiP. [102]. During ER stress (such as ER Ca^2+^ depletion), BiP dissociates followed by prolonged Ca^2+^ signaling into mitochondria via IP3Rs [102]. Studies on neuronal PC6.3 cells expressing N-terminal huntingtin fragment proteins with 120 polyglutamine repeats found decreased expression of S1R [84]. Additionally, Hyrskyluoto et al. also observed administration of Sig-1R agonist, PRE084, improved the survival of cells and helped to ameliorate the deleterious effect caused by the PolyQ repeats. PRE084 increased the levels of cellular antioxidants such as SOD1, SOD2, thioredoxin 2 by activating the NF-κB pathway. This shows Poly Q mediated HD toxicity is via S1R expression which controls NF-KB mediated upregulation of antioxidants and decreased ROS levels. Interestingly, in striatum of YAC128 HD mice (12 months) and in the HD patients, an upregulated S1R expression was observed [103]. S1R, under a physiological condition, plays a vital function in neurotransmission and synaptic plasticity through modulation of the NMDA receptor (NMDAR) activity inducing hippocampal long-term potentiation (LTP) [80]. However, under a pathophysiological condition as observed in HD, activation of NMDARs causes enhanced calcium influx resulting in excitotoxicity and neuronal death. Studies show that S1R facilitates NMDA receptor signaling and neurotransmission in hippocampal neurons by inhibiting Ca^2+^-activated SK channels. Thus, calcium dysregulation played a significant role controlling the HD symptoms.

Besides the above-described MAM proteins, which are significantly involved in the development of HD pathology, Table 2 describes MAM proteins that influence HD pathology.

## 5. Therapeutic Intervention for HD Targeting through ER Stress Pathways

Dysbiosis in the endomembrane system containing ER, Golgi apparatus, lysosome, and vesicle, together, leads to cellular imbalance as observed in many neurological disorders including HD. Despite its monogenic origin, HD has very complex pathophysiology. As it affects several signaling pathways, it is challenging in targeting particular pathways effectively. Currently, there is no cure for HD. However, several strategies have been tried in search of effective therapy. Recently, two different experimental approaches (knock down of mHtt and blocking cellular toxicity) have been implemented. RNA based targeted knockdown via siRNA or shRNA showed lasting reduction in mutant HTT protein in striatum [104,105]. Southwell et al. utilized allele-specific Antisense oligonucleotides targeting HD-SNPs that selectively suppress mutant HTT in a HD mouse model. The treatment resulted in reduced cognitive and behavioral impairments in the HD mice [106]. In transgenic HD (tgHD) minipig model, adeno-associated virus (AAV) mediated delivery of miRNA against HTT mRNA (AAV5-miHTT) significantly reduced both human mutant huntingtin mRNA and protein in all the brain regions [107]. Additionally, CRISPR/Cas9 based therapy also showed promising results. Allele-specific CRISPR/Cas9-mediated gene editing enabled permanent suppression of endogenous mHTT expression in the striatum of mHTT-expressing mice (HD140Q-knockin mice) [108]. Precise excision of CAG repeats through paired Cas9 nickase strategy HD patient-derived fibroblasts with varied numbers of CAG repeats opened another way toward effective therapy [109]. The main set back of all these methods are effective delivery systems for the successful treatment of HD patients. Significant research is also going on to circumvent this issue. An alternative strategy could be cell replacement therapy using human fetal-derived stem cells. Limited number of successful clinical trials found effective recovery and integration of medium spiny neurons (MSNs) after transplantation of developing MSNs taken from the fetal brain [110]. The other approaches, which focused on reducing cellular toxicity of mHtt by targeting different biologically important signaling proteins, also showed promising results. For example, targeting heat shock proteins [111], neurotrophic factors such as CNTF [112], HDACs [113], and proteasome activity [114] have significantly improved mHtt mediated toxicity. Pridopidine is a highly selective and potent Sigma-1 Receptor (S1R) agonist that has tremendous potential in treating HD, ALS, and other neurodegenerative diseases [115]. This is under global phase 3 clinical trial in HD [116].

### 5.1. Targeting UPR Sensors

**Figure 2:**
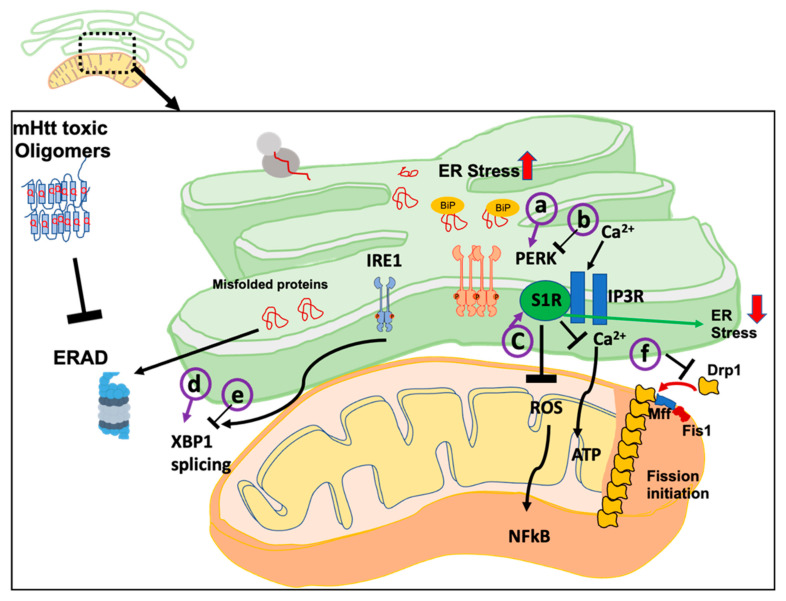
ER stress sensor proteins and MAM proteins as therapeutic targets in HD. Inhibition of ERAD by mHtt toxic oligomers can induce ER stress. ER stress sensors (like IRE1, PERK) sense proteostasis imbalance activates unfolded protein response pathways. The a, b, c, d, e & f indicate possible points of therapeutic intervention either at the level of ER stress sensor or MAM proteins.

Reduction in ER stress undoubtedly has beneficial effects on HD (Figure 2). PERK, one of the sensors of ER stress, is a very effective target for several neurodegenerative diseases. PERK inhibitors are found to be effective in HD, ALS, FTD, AD, and PD [49,117,118,119]. Recently, a PERK activator (MK-28) was found to be highly effective which can modulate eIF2alpha phosphorylation [69]. Several ERAD inhibitors are beneficial in treating protein misfolding diseases [120]. As already mentioned above, p97/VCP depletion improves HD pathogenicity via ERAD inhibition. ERAD inhibitor Eeyarestatin I has p97/VCP inhibitory group [121]. Thus, ERAD inhibition could potentially be effective in HD treatment. It is also found that VCP can interact with mHtt on mitochondria, enhancing mitophagy and cell death. A peptide based targeted inhibition was developed to inhibit this interaction [122]. Targeting other ER stress sensors has also been found to be beneficial. Selective silencing of XBP1 (a transcription factor activated by IRE1 upon ER stress) leads to a decrease in mHtt levels, along with reduced neuronal loss and improved cytotoxic effects of HD in HD transgenic mice. Mechanistically, it was found XBP1 deficient cells had upregulated Forkhead box O1 (FoxO1), indicating the autophagy-mediated clearance of mHtt [53]. The same group found a contrasting result with a more targeted gene therapy. Localized administration of an active form of XBP1 into the striatum of adult mice using adeno-associated vectors (AAVs) reduced mutant Huntingtin aggregation. The difference in the result is the use of model systems and the specific goal of the experiments. XBP1 deficiency was created in a mouse model. Knockout of UPR genes at embryogenesis stage might trigger other compensatory pathways such as autophagy as a rescue pathway [123]. From a mechanistic point of view, transgenic line studies provide insights into how UPR deficiency can be tackled by other protein homeostatic networks that are going to have beneficial effects in HD condition. With respect to therapeutic strategy, localized administration of an active form of XBP1 is more important, which actually showed the reduction in the aggregates. These two studies are tremendously important in making therapeutic strategies. One study has pointed out, on targeting the UPR pathway during the development of knockout mice, a probable influence on an overall change in ER homeostasis [53]. In contrast, targeted delivery is more important and mechanistically more successful in the context of targeting specific pathways [124].

Reduction in ER stress is protective in HD and can be accomplished, for example, with the use of chemical chaperones [125,126]. As mentioned above, p97/VCP depletion is an important factor in HD pathogenicity, through inhibition of ERAD and development of ER stress [10]. It was recently reported that VCP interacts with mHtt on mitochondria, enhancing mitophagy and cell death; a peptide was developed to inhibit the interaction [122]. Like the inhibition of ERAD, previous studies also reported inhibition of proteasome activity via expanded PolyQ aggregates and its effect on cell survivability [127,128,129]. Verhoef et al. found soluble PolyQ proteins with degradation signals can easily be degraded, irrespective of its length of PolyQ repeat. However, the aggregated condition, alone or with co-aggregated proteins, are resistant to degradation via proteasome [127]. Later, a detailed study by the Goldberg group demonstrated that eukaryotic 26S and 20S proteasomes failed to cut within stretches of 9–29 Q residues in peptides. Moreover, the occasional failure of these undegradable sequences may interfere with proteasome activity. A recent study also found PolyQ aggregates are able to sequester other co-chaperones into aggregates, resulting in an imbalance of protein homeostasis leading to cellular toxicity [130].

As chemical chaperones improve ER function and ER stress tolerance, they have also been used in several other neurodegenerative diseases as therapeutic interventions. TUDCA (taurine-conjugated ursodeoxycholic acid), a chemical chaperon, alleviated ER stress and helped to reduce neuropathy in the striatum and improve motor and sensory symptoms [126].

Natural compounds such as curcumin, sulforaphane, and resveratrol with vitamin D3 were shown to have potential beneficial effects in neurodegenerative diseases [131,132]. Deficiency of 25-hydroxy vitamin D, the major circulating form of vitamin D, is associated with an increased risk of age-related neurodegenerative diseases such as AD, PD, HD. It has been reported that vitamin D deficiency in patients manifests the HD symptoms [133]. Interestingly, in our unpublished data, we found vitamin D3 combated stress pathways in 3-nitropropionic acid induced mouse models of HD. Thus, vitamin supplementation could be useful for treating complex diseases such as HD. A recent study using *C. elegans* reported that vitamin D promotes protein homeostasis and longevity via stress response pathway genes, including ER stress sensor (*skn-1*, *ire-1*, and *xbp-1*) [134]. Thus, more investigation is required to understand the mechanism of how vitamin D interacts with different stress pathways including ER stress. It will further be helpful to mechanistically understand its therapeutic potential to cure HD.

### 5.2. Targeting MAM Proteins

Due to its involvement in maintaining ER mitochondrial health, S1R was found to be an excellent target for ameliorating symptoms in HD (Figure 2). During ER stress, there is an enhancement of S1R-dependent mitochondrial bioenergetics [135]. It indicates controlling S1R could be beneficial in controlling the homeostasis of two interconnected organelle. Earlier experiments found that administration of S1R agonist PRE-084 had a neuroprotective role by restoring S1R deficit and decreased ROS level via NF-kB signaling [84]. Another agonist, pridopidine, was found to be effective in different studies involving R6/2 and Yac128 HD mouse models [136,137,138]. It improves motor performance and survival of the striatum neurons, mainly through regulation of calcium homeostasis [103]. This drug was further tested in a large population (phase 2 trial) for its safety and efficacy in patients with Huntington’s disease [84,115,139]. It is now moving toward phase 3 trial [116].

## 6. Conclusions

Undoubtedly, HD is one of the complex disorders in which ER proteostasis is perturbed (Table 3). Additionally, ER mitochondrial signaling is significantly involved in the development of HD pathophysiology. While UPR pathways and ER-mitochondria crosstalk have been extensively characterized, it is still unclear why integrated stress signaling failed to cope with stress in specific cells, such as striatum neurons, during HD. Nevertheless, targeting ER stress pathways in HD remains an attractive approach to prevent cell death, and it will be one of the ways toward effective treatments against this disorder. Recent success targeting UPR pathways, specifically PERK, opens up a new therapeutic intervention. However, there is a requirement for more mechanistic study to understand the activity of PERK more precisely. Irrespective of that, the goal is to activate the PERK at an optimal point that will help to alleviate the HD toxicity. Similarly, S1R, the MAM protein, might be a promising target for HD treatment. Focus should also be implemented by introducing other compounds such as vitamin D, known for its beneficial effects in other neurodegenerative diseases.

## Figures and Tables

**Table 1:** ER stress sensors and HD.

Sensor Proteins	Functions of Proteins during HD	References
IRE1	Inhibition of autophagy via IRE-1-TRAF2 may impair autophagic clearance of mHtt aggregates leading to neuronal degeneration.	Lee et al., 2011
	Overexpression of the ubiquitin-specific protease-14 (Usp14) reduces cellular aggregates in mutant Htt-expressing cells. Overexpression of Usp14 in turn was able to inhibit phosphorylation of IRE1α in mutant Htt-overexpressing cells and to protect against cell degeneration and caspase-3 activation.	Hyrskyluoto et al., 2014
PERK	ERK/eIF2α phosphorylation is involved in polyQ72-induced LC3 conversion, which is a prerequisite for autophagy in the expression of Huntington’s disease. It acted as a cellular defence that inhibited ER-stress-mediated cell death.	Kouroku et al., 2007
	This study proposed that use of salubrinal can reduce ER stress and counteract cell death caused by the mutant huntingtin proteins. Here increased phosphorylation of eukaryotic translation initiation factor2 subunit-α (eIF2α) by the salubrinal has shown to be neuroprotective, thereby influencing the PERK pathway.	Reijonen et al., 2008
	ERAD substrate accumulation and the related ER stress are not dependent on the presence and concentration of large visible aggregates but arise when Htt is in a monomeric or oligomeric state. Eventually all the three UPR sensors are activated including PERK.	Leitman et al., 2013
	It reveals that cultured striatal cells and murine brain striatum have remarkably low levels of phosphorylation of translation initiation factor eIF2α. eIF2α phosphorylation was elevated in a striatal cell line stably expressing pathogenic huntingtin and in brain sections of Huntington’s disease model mice.	Leitman et al., 2014
	PromISR-6 is a potent GBZ analogue, has the capability to prolong the phosphorylation time of eIF2α and protein translation inhibition, reducing mutant Htt aggregates and increasing survival in an HD cellular model, apparently by activating autophagy	Sundaram et al., 2019
	Ganz et al. developed a potent small molecule PERK activator MK-28, which showed improvement in cellular and mice models of HD. It rescued cells from undergoing ER-stress induced apoptosis. Also, it significantly improved motor and executive functions and delayed death onset in the HD induced mice model.	Ganz et al., 2020
	The level of eIF2α phosphorylation is significantly lower in the striatal cells. Interestingly, the presence of pathogenic mtHtt increased the eIF2 alpha phosphorylation in STHdh Q111/111 cells and in the murine striatum	Ma et al., 2020
ATF6	ATF6α has also been demonstrated to mediate Rheb expression, and both proteins cooperate to maintain the survival of postmitotic neurons. The ATF6α/Rheb pathway is altered in Huntington’s disease as the decrease in ATF6αprocessing is accompanied by a decrease in the accumulation of Rheb. These alterations correlate with the aberrant accumulation of cell cycle re-entry markers in post-mitotic neurons which is accompanied by death of a subset of neurons. It also leads to, compromised execution of the ER-stress response which may contribute to the pathogenesis of HD.	Fernandez et al., 2011
	They have studied the effect of TD-induced neurodegeneration and seen that ER stress markers, such as GRP78, XBP-1, CHOP, ATF-6, phosphorylated eIF2α, and cleaved caspase-12 have increased rapidly.	Wang X et al., 2020

**Table 2:** Involvement of MAM proteins in HD.

MAM Proteins	Functions of Proteins during HD	Reference
Drp 1	Aberrant Drp1-mediated mitochondrial fragmentation within the striatum of HD mutant mice, forces mitochondria to place far away from the ER disrupting the ER-mitochondria association and therefore causing changes in Ca^2+^ efflux and an excessive production of mitochondria superoxide species.	Cherubini et al., 2020
PERK	There is a strong ER stress induction and UPR activation in striatal neurons expressing mHtt. PERK pathway is strongly downregulated in striatal neurons compared to other cell types and brain regions in WT mice and is upregulated in HD. PERK activation is an effective and selective strategy to reduce ER stress, re-establish ER homeostasis and increase the survival of cells and mice expressing mHtt.	Ganz et al., 2020
RyR 2	RyR channels were oxidized, PKA phosphorylated, and leaky in brain, heart, and diaphragm both in patients with HD and in a murine model of HD. Defective RyR function has been reported in HD, leading to elevated intracellular Ca^2+^ levels and reduced endoplasmic reticular Ca^2+^ stores in R6/2 striatal and cortical neurons .	Dridi et al., 2020
ATAD3A	In HD, ATAD3A forms oligomers which bridge Drp1-mediated mitochondrial fragmentation and mtDNA instability, leading to impaired mitochondrial biogenesis and neurodegeneration.	Zhao et al., 2019
Fis 1	Heart is highly susceptible to the effects of mHTT. Drp1 hyperactivation through its interaction with Fis1 plays a role in the pathogenesis of polyglutamine induced cardiac mitochondrial dysfunction. Inhibitor for Drp1/Fis1 interaction, reduced pathological mitochondrial fission in mouse and human cell models of HD.	Joshi et al., 2018
CHOP	mHtt expressing striatal cells also exhibited enhanced ER stress in response to serum deprivation, indicated by upregulation of CHOP. The upregulation of CHOP can also downregulate Bcl2 and upregulate pro-apoptotic Bax and Bak. Increase in CHOP expression leads to oxidative stress response in the cells.	Zhou et al., 2018
DISC1	Disturbance of DISC1-PDE4 interactions and behavioral changes through aggregation of DISC1 in HD.HTT forms a ternary protein complex with the scaffolding protein DISC1 and cAMP-degrading phosphodiesterase 4 (PDE4) to regulate PDE4 activity. Aggregation of DISC1 is significantly accelerated by the presence of mutant HTT, and they recapitulate the PolyQ pathology of HD. Cross-seeding of mutant HTT and DISC1 and the resultant changes in PDE4 activity may underlie the pathology of a specific subset of mental manifestations of HD.	Tanaka et al., 2017
OPA 1	Integrity of the mitochondrial cristae is compromised in striatum and cortex of the HD mice and that this is most likely caused by impaired OPA1 oligomerisation. Mutant huntingtin reduces OPA1 gene expression and promotes OPA1 cleavage.	Hering et al., 2016
VDAC 1	The expression of mHtt may promote VDAC closing, a situation known to be accompanied by changes of VDAC selectivity toward cations. This, in turn, may influence metabolite exchange between mitochondria and cytoplasm. Decreased conductance of the open state and decreased voltage-dependence reflect VDAC functional changes occurring in cells with expression of mHtt.	Karachitos et al., 2016
PINK1	PINK1 overexpression alleviated mitochondrial spheroid formation. Mitophagy is altered in the presence of mHtt and that increasing PINK1/Parkin mitochondrial quality control pathway may improve mitochondrial integrity and neuroprotection in HD.	Khalil et al., 2015
mTOR	Htt promotes signaling by mTORC1 [mechanistic target of rapamycin (mTOR) complex 1] and that this signaling is potentiated by poly-Q–expanded Htt. Htt promotes amino acid mediated mTORC1 signaling that stimulated the interaction of Htt and the guanosine triphosphatase (GTPase) Rheb (a protein that stimulates mTOR activity), and that Htt forms a ternary complex with Rheb and mTOR.	Pryor et al., 2014
Sigma- 1 Receptor	Sig-1R protein levels were decreased in mutant huntingtin-expressing cells. Partial co-localization of Sig-1R with aggregates in mutant huntingtin protein-expressing cells were observed, suggesting that the Sig-1R may be redistributed or delocalized in these cells.	Hyrskyluoto et al., 2013
IRE1	Regulates mutant huntingtin (HTT) clearance and most studies have shown that IRE1 can control autophagy levels by recruiting the adaptor protein TRAF2 and activating MAPK8 (mitogen-activated protein kinase 8)/JNK	Fouillet et al, 2012; Paolo Remondelli and Maurizio Renna 2017
IP3R	Mutant huntingtin bind to the carboxy-terminal region of the type 1 inositol 1,4,5-trisphosphate receptor (IP3R1) and causes sensitization of IP3R1 to activation by IP3 in planar lipid bilayers and in neuronal cells. InsP3R1 association of ER stress chaperone protein GRP78 is impaired in Huntington’s disease R6/2 model mice, resulting in misregulation of InsP3R1 gating. Impairment of IP3R1 function promotes mitochondria-dependent cell death in the model mice by producing negative effects on mitochondrial Ca^2+^, the membrane potential and depletion in mitochondrial ATP production.	Higo et al., 2012
Mfn 1	Levels of Mfn 1 was decreased in mutant Htt neurons, confirming the presence of abnormal mitochondrial dynamics in the cortex of HD patients, and may contribute to neuronal damage in HD patients	Shirendeb et al., 2011
Fis 1	FIS1 was highly associated with late-stage HD rather than the pre-symptomatic stage. Fis1 recruits Drp1 under cell stress in numerous neurodegenerative disease models. In HD, the balance between fusion and fission is aberrantly shifted toward fission, which is associated with increased levels of Fis1 mRNA and decreased mitofusins in striatal and cortical regions, leading to mitochondrial fragmentation.	Shirendeb et al., 2011; Xiang et al., 2020
Mfn 2	Mfn2 can directly interact with the N-terminus of mutant Htt, suggesting that Htt can interfere directly with the function of Mfn2 in the promotion of mitochondrial elongation and in the regulation of the apoptotic function of Bax. Mutant Htt, also interfere with the extramitochondrial functions of Mfn2, leading to alterations in the shape of the ER, in the ER levels of Ca2 þ , and last but not least in the tethering of mitochondria to the ER.	Wand et al., 2009; de Brito OM and Scorrano L (2008)
ITPR 1/3	The polyQ-Htt can bind ITPR1 with high affinity, sensitizing the receptor activity by IP3. Blocking the Htt–ITPR1 interaction in vivo was shown to regulate the abnormal calcium signaling in response to glutamate, protecting the neurons from death, and improving motor coordination	Tang et al., 2009
PP2A	Several holoenzyme complexes of PP2A have been isolated from a variety of tissues and have been extensively characterized. PP2A consists of a regulatory subunit termed as PR65. The structure of PR65 is unusual, since it is entirely composed of 15 tandem repeats of a 39-amino-acid sequence, termed as HEAT (huntingtin elongation A subunit TOR, where TOR is target of rapamycin) motif. HEAT sequence is present in huntingtin protein, an elongation factor required for protein synthesis and the TOR kinase. These domains play a role in a variety of interactions between proteins.	Hiroki Takano and James F Gusella 2002
Grp 75	The interaction between calcium channel IP3R and VDAC1 in the outer mitochondrial membrane is strengthened by Grp75. Compared with Wild type mice, a significant decrease in Grp75 levels was observed in R6/1mice at 12 and 20 weeks of age.	Cherubini et al., 2020

**Table 3:** Proteins or pathways affected in HD.

HD Conditions	Regulation	Reference
Primary neurons of mice showed proteasome dysfunction- and ER stress-induced neuronal cell death, which gives important neuropathological alterations in HD	Overexpression of IRE1 mediated TRAF-2ASK1 and JNK signalling activation	Nishitoh et al., 2002
STHdhQ7/7 striatal cells were treated with tunicamycin, a well-known inducer of ER stress	Bip, CHOP, Herpud1 upregulation,Overexpression of Rrs1, ER stress related marker	Carnemolla et al., 2009
Expanded PolyQ induces cell death in neuronal PC6.3 cells	Downregulation of NF kB via IRE1-TRAF2 ER stress pathway	Reijonen et al., 2008
Accumulation of mHtt in HD mouse model and HD patient striatal tissues was seen	Upregulation of p-IRE1 (ER stress marker) and p62 (Autophagy marker)	Lee et al., 2011
Studying HD pathogenesis through a Fly model (Htt-Q128 fly, a HD model expressing Htt-Q128)	Downregulation of IRE1 rescued rough eye phenotype	Lee et al., 2011
Full-length mHtt transgenic mouse strain YAC128 was used in the study	Silencing XBP-1 expression reduces neuronal loss in the striatum and improves motor performance. It in turn increases expression Foxo1 and autophagy levels	Vidal et al., 2012
Soluble Oligomeric PolyQ expanded HD was expressed in HEK 293 cells in the presence of Htt96Q (containing exon 1 with an expanded tract of 96 glutamines) and in striatal cells	Inhibit ERAD and induces high expression 3 branches of ER stress (IRE1, PERK, and ATF6)	Leitman et al., 2013
Huntingtin-exon 1 aggregates in vitro and in HeLa cells were used in the study	The study identified disaggregase activity of valosin-containing protein (VCP/p97) that can detangle the accumulations of toxic proteins, such as Huntingtin-exon 1 aggregates which can reduce ER stress	Ghosh et al., 2018
Stable MC3T3-E1 cell lines overexpressing exon 1 of either the wild type Huntingtin protein (wtHTT), which contains a string of 23 Gln residues, or a disease-causing mutant Huntingtin (mHTT), which contains 145 Gln’s were used for the study	Activated IRE1 degrades Blos1 mRNA it enhances endosomal microautophagy pathway, protects cells from toxicity of mHtt	Bae et al., 2021
Presence of mHtt in STHdhQ111/111 and murine striatum	Phosphorylation of eIF2alpha is low in striatal cells, and presence of mHtt increase the expression of eIF2alpha	Ma R-H et al., 2020; Leitman et al., 2020
Cellular HD model (a murine striatal cell line) with knock-in of a full-length PolyQ-expanded mHtt (STHdhQ111/111), was used in the study. HD mouse model, the R6/2 with 160 CAG repeats were found to undergo recovery after PERK activation	MK-28 is an activator of PERK. MK-28 significantly improved motor and executive functions and delayed death onset in R6/2 mice, showing no toxicity. PERK activation is beneficial and can help treat aggressive model of HD	Ganz et al., 2020
R6/1 and R6/2 animals (B6CBA-Tg(HDexon1)61Gpb) with expansion of 115 and 150 glutamines respectively. Brain samples from HD patients and age-matched controls were also used	ATF6 alpha is impaired in both animal models of the disease as well as in the brains of HD patients. These alterations are accompanied by a decrease in Rheb. The decreased accumulation of both proteins correlates with the re-entry of neurons into the cell cycle	Fernandez et al., 2010
R6/1 mice model with close to 150 glutamine repeats were used	DREAM inhibition markedly enhance ATF6 expression in the hippocampus and that it might contribute to a delay in memory decline in HD mice.	Hurtado et al., 2018
Thiamine (vitamin B1) deficiency (TD) induced neurodegeneration study. Human neurons differentiated from induced pluripotent stem (iPS) cells was used	The study showed upregulation in different proteins such as GRP78, XBP-1, CHOP and ATF-6	Wang et al., 2019
Post-mortem neostriatal tissue specimens from 35 adult-onset HD patients was used to carry out the study on both mitochondrial loss and altered mitochondrial morphogenesis with increased mitochondrial fission and reduced fusion in HD.	Mitochondrial dysfunction plays a critical role in the pathogenesis of HD. Increased expression of DRP1 protein and Decreased expression of MFN1 can lead to neuronal loss and disease progression in HD patients	Kim et al., 2010
PC6.3 neuronal cells were used to study that compounds influencing Sig-1R may constitute promising targets for future drug developments in HD.	Sig-1R agonist, PRE084 increases cell survival and counteracts the deleterious effects caused by N-terminal mutant huntingtin proteins in neuronal PC6.3 cells. Sig-1R expression is increased which enhance the levels of cellular antioxidants by activating the NF-kB pathway that is compromised by the expression of mutant huntingtin proteins	Hyrskyluoto et al., 2013
In vitro and In vivo mouse model studies were conducted to see the beneficial effects of S1R during HD	Increase in the levels of S1R through pridopidine in the striatal region exerts long-term beneficial effects on HD. Pridopidine improved motor performance and prolonged survival of R6/2 HD mice and exerted neuroprotective effects in a mouse striatal knock-in cellular model of HD	Ryskamp et al., 2017

## Data Availability

Not applicable.

## Abbreviations

HD: Huntington’s Disease; CAG trinucleotide repeat: Cytosine-Adenine-Guanine; mtHTT: Mutant Huntingtin; PolyQ: Poly Glutamine; Hsp40: Heat Shock Protein 40; ER: Endoplasmic Reticulum; ERAD: Endoplasmic Reticulum Associated Degradation; UPR^ER^: Unfolded Protein Response of ER; IRE1: Inositol Requiring Protein 1; XBP-1: X-Box Binding Protein 1; RIDD: Regulated Ire1-Dependent Decay; PERK: Protein Kinase RNA-like ER Kinase; eIF2 alpha: Eukaryotic Translation Initiation Factor 2 alpha; ATF4: Activation of Transcription Factor 4; *C. elegans*: *Caenorhabditis elegans*; C/EBP—CHOP: CCAAT Enhancer Binding Protein Homologous Protein; ATF6: Activating Transcription Factor 6; S1P: Site 1 Protease; S2P: Site 2 Protease; Grp78: Glucose Regulated Protein 78; BiP: Binding Immunoglobulin Protein; PDI: Protein Disulphide Isomerase; Cdc48p/p97: Cell Division Protein 48; TRAF2-ASK1: Tumor Necrosis Factor Receptor Associated Factor2—Apoptosis Signal Regulating Kinase1; JNK: C-Jun N-Terminal Kinase; HERPUD1: Homocysteine Inducible ER Protein with Ubiquitin-Like Domain 1; RRS1: Ribosome Biogenesis Regulator 1 Homolog; NFkB: Nuclear Factor Kappa Light Chain Enhancer of Activated B Cells; FoxO1: Forkhead Box O1; P97/VCP: Valosin-Containing Protein; MA: Macro Autophagy; LEs: Late Endosomes; MTOC: Microtubule Organizing Center; BORC: BLOC1-Related Complex; Atg5: Autophagy Related 5; LC3-I: Microtubule-Associated Protein 1A/1B-Light Chain 3; LC3-II: LC3-Phosphatidylethanolamine Conjugate; Rheb: Ras Homolog Enriched in Brain; NSC: Neuronal Calcium Sensor; DREAM: Downstream Regulatory Element Antagonist Modulator; TD: Thiamine Deficiency; UFD1: Ubiquitin Recognition Factor in ER Associated Degradation 1; Gp78: Tumor autocrine motility factor receptor; E3 ligase: ER Membrane Anchored Ubiquitin Ligase; DTT: Dithiothreitol; MAM: Mitochondria Associated-ER Membrane; S1R: Sigma 1 Receptor; Drp 1: Dynamin-Related Protein 1; FIS 1: Mitochondrial Fission 1 Protein; OPA 1: OPA 1 Mitochondrial Dynamin-Like GTPase; Mfn 1: Mitofusin 1; ROS: Reactive Oxygen Species; IP3R: Inositol Triphosphate Receptor Type 3; Ca^2+^: Calcium ions. SOD 1/2: Superoxide Dismutase ½; NMDAR: N-methyl-D-aspartate Receptor; LTP: Long-term Potentiation; siRNA: Small Interfering RNA; shRNA: Short or Small Hairpin RNA; tgHD: Transgenic HD; AAV: Adeno-Associated Virus; MSN: Medium Spiny Neurons; CNTF: Ciliary Neurotrophic Factor; HDAC: Histone Deacetylases; ALS: Amyotrophic Lateral Sclerosis; AD: Alzheimer’s disease; FTD: Frontotemporal Dementia; TUDCA: Taurine-Conjugated Ursodeoxygolic Acid.
